# Orai2 Modulates Store-Operated Ca^2+^ Entry and Cell Cycle Progression in Breast Cancer Cells

**DOI:** 10.3390/cancers14010114

**Published:** 2021-12-27

**Authors:** Jose Sanchez-Collado, Jose J. Lopez, Carlos Cantonero, Isaac Jardin, Sergio Regodón, Pedro C. Redondo, Juan Gordillo, Tarik Smani, Gines M. Salido, Juan A. Rosado

**Affiliations:** 1 Cellular Physiology Research Group, Department of Physiology, Institute of Molecular Pathology Biomarkers (IMPB), University of Extremadura, 10003 Caceres, Spain; josesc@unex.es (J.S.-C.); jjlopez@unex.es (J.J.L.); carloscantonero@unex.es (C.C.); ijp@unex.es (I.J.); sergio@unex.es (S.R.); pcr@unex.es (P.C.R.); gsalido@unex.es (G.M.S.); 2 Pathology Service, Hospital de Merida, 06800 Merida, Spain; juan.gordillo@salud-juntaex.es; 3 Department of Medical Physiology and Biophysics, University of Seville, 41004 Seville, Spain; tasmani@us.es; 4 Group of Cardiovascular Pathophysiology, Institute of Biomedicine of Seville, University Hospital of Virgen del Rocio, University of Seville, CSIC, 41004 Seville, Spain

**Keywords:** Orai2, breast cancer, store-operated calcium entry, NFAT, cell cycle

## Abstract

**Simple Summary:**

Breast cancer shows a high heterogeneity which accounts for cancer subtype aggressiveness. Store-operated calcium entry (SOCE) is significantly remodeled in breast cancer cells and supports a variety of cancer hallmarks. Here we show that breast cancer cells from different subtypes exhibit a variable Orai1:Orai2 expression ratio. In cells with a high Orai2 expression, Orai2 modulates the magnitude SOCE and support Ca^2+^ oscillations in response to agonists, thus modulating the activation of the transcription factors NFAT1 and NFAT4. Furthermore, Orai2 plays a relevant role in cell cycle progression and apoptosis resistance.

**Abstract:**

Breast cancer is a heterogeneous disease from the histological and molecular expression point of view, and this heterogeneity determines cancer aggressiveness. Store-operated Ca^2+^ entry (SOCE), a major mechanism for Ca^2+^ entry in non-excitable cells, is significantly remodeled in cancer cells and plays an important role in the development and support of different cancer hallmarks. The store-operated CRAC (Ca^2+^ release-activated Ca^2+^) channels are predominantly comprised of Orai1 but the participation of Orai2 and Orai3 subunits has been reported to modulate the magnitude of Ca^2+^ responses. Here we provide evidence for a heterogeneous expression of Orai2 among different breast cancer cell lines. In the HER2 and triple negative breast cancer cell lines SKBR3 and BT20, respectively, where the expression of Orai2 was greater, Orai2 modulates the magnitude of SOCE and sustain Ca^2+^ oscillations in response to carbachol. Interestingly, in these cells Orai2 modulates the activation of NFAT1 and NFAT4 in response to high and low agonist concentrations. Finally, we have found that, in cells with high Orai2 expression, Orai2 knockdown leads to cell cycle arrest at the G0-G1 phase and decreases apoptosis resistance upon cisplatin treatment. Altogether, these findings indicate that, in breast cancer cells with a high Orai2 expression, Orai2 plays a relevant functional role in agonist-evoked Ca^2+^ signals, cell proliferation and apoptosis resistance.

## 1. Introduction

Store-operated Ca^2+^ entry (SOCE) is a major mechanism for Ca^2+^ influx in non-excitable cells regulated by the filling state of the agonist-releasable intracellular Ca^2+^ stores [1]. The key elements of SOCE include the endoplasmic reticulum (ER) Ca^2+^ sensor STIM1, and its homolog STIM2, and the Ca^2+^ release-activated Ca^2+^ (CRAC) channels, predominantly comprised of Orai1 [2,3]. Orai1 has two homologs, Orai2 and Orai3, whose contribution in the native CRAC channels has remained elusive. Nevertheless, the generation of Orai isoform-specific knockout cell lines in mice has provided strong evidence supporting that heteromerization of Orai isoforms fine tunes CRAC-mediated Ca^2+^ signaling, thus matching the Ca^2+^ responses to the strength of agonist stimulation [4,5]. According to this, Orai2 and Orai3 has been reported to modulate the magnitude of SOCE in a variety of cell types, including HEK293 cells [4,5], human Hs27 fibroblasts [5], the chondrocyte cell line OUMS-27 [6], Jurkat T cells [7], primary ameloblasts [8] and human neuroglioma derived cells [9], as well as in Ora2-deficient mice [5]. This phenomenon is probably mediated because Orai2 and Orai3 have a greater sensitivity to Ca^2+^-dependent inactivation than Orai1 [10,11]. By contrast, Orai2 has been reported to support SOCE in different cell types, such as HL-60 acute myeloid leukemia cells [12], bone marrow neutrophils [13] and gastric cancer cells [14]. Unlike Orai3, Orai1 and Orai2 share some biochemical features such as their sensitivity to pH, so that Orai1 and Orai2 currents are inhibited by acidification and enhanced by alkalization, while those of Orai3 are unaffected [15,16].

In addition to their implication in different physiological processes, Orai2 and Orai3 are also involved in a variety of pathophysiological events, including cancer. Orai2 and Orai3 have been increasingly identified as relevant in the development of different types of cancer, including leukemia, prostate cancer, non-small cell lung adenocarcinoma and breast cancer [12,17,18,19,20].

Breast cancer is among the most commonly occurring cancer in women and one of the leading causes of cancer death in western countries. Considering the histology and molecular profile, breast cancer is a heterogeneous disease. Different subtypes have been identified according to the gene expression profile, including luminal A, luminal B, HER2 and basal or triple negative subtypes [21]. This classification reflects the different aggressiveness and metastatic potentials of the different breast cancer subtypes. The aggressiveness is greater for triple negative and HER2 subtypes and smaller for the luminal cancer types [22]. SOCE has been reported to be significantly remodeled in breast cancer cells and the integration of gene expression and functional studies has recently led to the characterization of abnormal Ca^2+^ signaling in cells from the different breast cancer molecular subtypes. Concerning CRAC channels, Orai1 has been reported to be overexpressed in all the breast cancer cell subtypes investigated, and breast cancer cell biology is strongly dependent on this channel [19,20,23,24]. Orai3 is selectively overexpressed in luminal A breast cancer cells, where SOCE, as well as the development of different cancer features including cell migration and proliferation, strongly depends on Orai3 [19,25]. However, the expression profile and functional role of Orai2 in breast cancer cells remains unclear. Here, we provide evidence for a different expression of Orai2 and a different relative Orai1:Orai2 expression profile in different breast cancer cell lines. In cells with a low Orai1:Orai2 expression ratio, Orai2 plays a relevant role in modulating the magnitude of SOCE and sustaining Ca^2+^ oscillations in response to agonist stimulation, which, in turn, modulates agonist stimulation of NFAT1 and NFAT4 nuclear translocation. Furthermore, our results indicate that Orai2 is required for cell cycle progression and apoptosis resistance in cells with a high Orai2 expression. Therefore, these findings strongly support an important role for Orai2 in the pathophysiology of breast cancer subtypes with a high Orai2 expression profile.

## 2. Results

### 2.1. Orai1 and Orai2 Relative Expression in ER+ and Triple Negative Breast Cancer Cells

According to the Cancer Genome Atlas (TCGA) database and analyzed using UALCAN (http://ualcan.path.uab.edu, accessed on 20 November 2021) [26], Orai1 and Orai2 mRNA expression is significantly higher in breast cancer tissues than in normal tissues (Figure S1), and this difference was consistent across groups stratified by cancer subtypes (luminal, HER2 and triple negative; Figure S1b,d; *p* < 0.05). According to these data, Orai2 upregulation in cancer cells was greater than that of Orai1 (1.2 vs. 1.7 for Orai1 and Orai2, respectively; Figure S1a,c).

**Figure 1:**
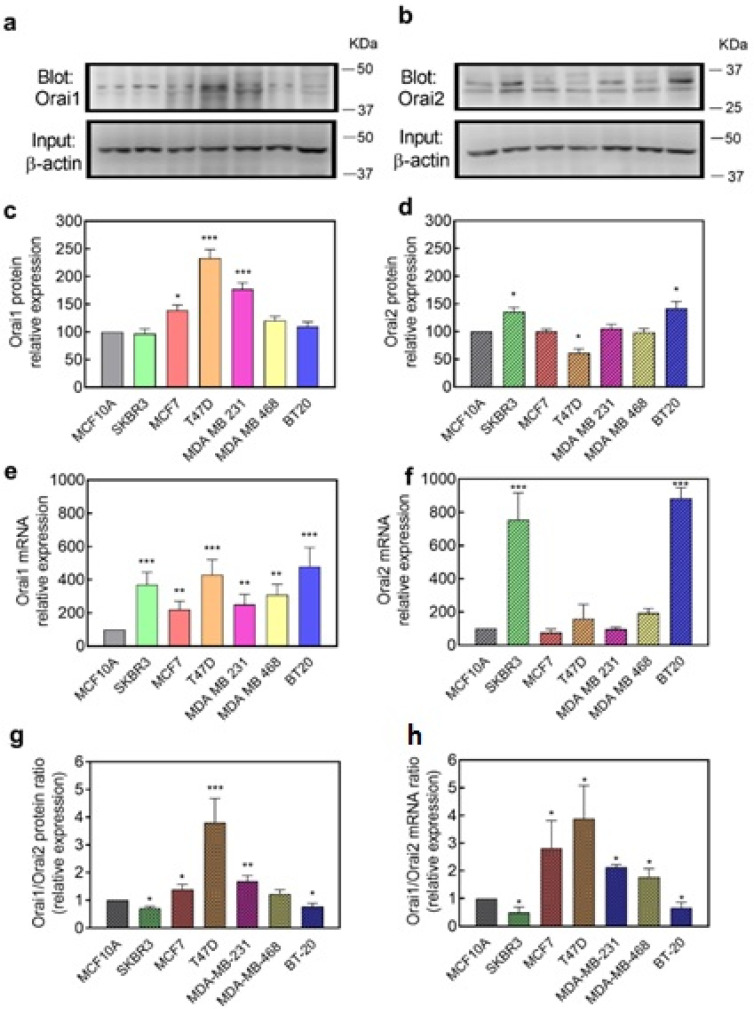
Orai1 and Orai2 mRNA and protein expression in breast cancer cell lines. Cells from the non-tumoral MCF10A cell line, as well as the HER2+ SKBR3, ER+ MCF7 and T47D and triple negative MDA-MB-231, MDA-MB-468 and BT20 breast cancer cells lines were lysed, and 50 μg of proteins each were loaded in the same gels. Whole cell lysates were subjected to 10% SDS-PAGE and Western blotting with the anti-Orai1 antibody (**a**) or anti-Orai2 antibody (**b**), as described in “Material and Methods”. Membranes were reprobed with anti-β-actin antibody for protein loading control. Molecular masses indicated on the right were determined using molecular-mass markers run in the same gel. Blots are representative of four to six separate experiments and were analyzed using Image J software, and densitometric ratios to corresponding actin were calculated. (**c**,**d**) Bar graphs represent Orai1 (**c**) or Orai2 (**d**) protein expression presented as mean ± SEM as expressed as percentages of the non-tumoral cell line MCF10A. (**e**,**f**) RT-qPCR expression analysis of Orai1 (**e**) and Orai2 (**f**) mRNA transcripts in the cell lines indicated above. Values were normalized to β-actin expression and represented as mean expression relative to MCF10A ± S.E.M.; *n* = 4. (**g**,**h**) Bar graphs represent the Orai1/Orai2 expression ratio in the different cell lines at the protein (**g**) or transcript (**h**) level, represented as mean expression relative to MCF10A ± S.E.M. Data were statistically analyzed using Kruskal–Wallis test with multiple comparisons (Dunn’s test) (* *p* < 0.05, ** *p* < 0.01, and *** *p* < 0.001 as compared to MCF10A cells).

As Orai2 has been reported to modulate Ca^2+^ influx through CRAC channels primarily formed by Orai1 subunits, we first explored the expression of Orai1 and Orai2 at the transcript and protein level in non-tumor breast epithelial cells as well as in ER+, HER2 and triple negative breast cancer cells (TNBC). We chose MCF10A cells as non-tumor cells; SKBR3 as representative of HER2 breast cancer cells; two ER+ breast cancer cell lines: MCF7 and T47D; and three TNBC cell lines: MDA-MB-231, MDA-MB-468 and BT20 cells. Analysis of the expression of Orai1 and Orai2 at the protein level was carried out by Western blotting using specific antibodies. Data were normalized with the expression of β-actin and revealed a predominant expression of Orai1 at the protein level in T47D, MDA-MB-231 and MCF7 cells that was significantly greater than that observed in MCF10A cells (Figure 1a,c; *p* < 0.001). Concerning Orai2, analysis of its expression in these cells revealed a significantly greater expression of the protein in SKBR3 and BT20 as compared to MCF10A cells (Figure 1b,d; *p* < 0.05). In contrast, in T47D the expression of Orai2 was found to be significantly lower than that in non-tumor MCF10A (Figure 1b,d; *p* < 0.05).

Analysis of the expression of Orai1 and Orai2 at the transcript level was carried out by qRT-PCR and revealed a greater expression of Orai1 mRNA transcript in all the breast cancer cell lines investigated as compared to non-tumor MCF10A cells (Figure 1e; *p* < 0.01). As for the Orai2 protein expression, analysis of the expression of Orai2 at the transcript level revealed a significantly greater expression in SKBR3 and BT20 cells (Figure 1f; *p* < 0.001).

We have further analyzed the Orai1:Orai2 expression ratio in the cell types investigated. We cannot compare the expression of Orai1 and Orai2 in a given cell type as we are using different antibodies for both proteins, but the Orai1:Orai2 expression ratio might provide an estimation of the relative expression of both Orai isoforms between different cell types. The analysis of the Orai1:Orai2 expression ratio at the protein and transcript level indicates that T47D cells exhibit a greater Orai1/Orai2 expression ratio both at the protein and transcript level (Figure 1g,h; *p* < 0.001), while SKBR3 and BT20 show an Orai1:Orai2 expression ratio that is significantly smaller than that in non-tumor MCF10A cell (Figure 1g,h; *p* < 0.05). These findings indicate that there are significant differences in the relative expression of Orai1 and Orai2 between breast cancer cells which might handle Ca^2+^ entry in a different way.

### 2.2. Role of Orai2 in SOCE in Cells with High and Low Orai1:Orai2 Expression Ratio

**Figure 2:**
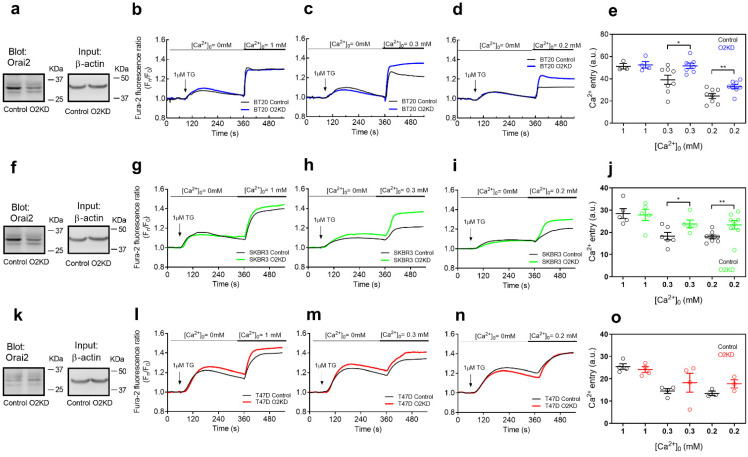
The functional role of Orai2 in store-operated Ca^2+^ entry in breast cancer. BT20 (**a**), SKBR3 (**f**) and T47D (**k**) cells were transfected with esiOrai2 (O2KD) or non-specific siRNA (Control; C). Forty-eight hours later cells were lysed and whole cell lysates were subjected to 10% SDS-PAGE and Western blotting with the anti-Orai2 antibody, as described in Section 4. Membranes were reprobed with anti-β-actin antibody for protein loading control. Molecular masses indicated on the right were determined using molecular-mass markers run in the same gel. Blots are representative of four to seven separate experiments. (**b**–**o**) BT20 (**b**–**d**), SKBR3 (**g**–**i**) and T47D (**l**–**n**) cells were transfected with esiOrai2 (O2KD) or non-specific siRNA (Control). Forty-eight hours later cells were loaded with Fura-2 and were continuously superfused with Ca^2+^-free HBSS or HBSS containing 1 mM, 0.3 mM or 0.2 mM Ca^2+^, as indicated. Cells were stimulated with 1 µM TG at 1 min (as indicated). (**e**,**j**,**o**) Quantification of TG-induced Ca^2+^ influx in BT20 (**e**), SKBR3 (**j**) and T47D (**o**) cells under the different experimental conditions (*n* = 3–8; n-values correspond to independent experiments). Scatter plots are represented as mean ± SEM and were statistically analyzed using Student´s *t*-test (* *p* < 0.05 and ** *p* < 0.01).

Next, we explored the role of Orai2 in TG-induced SOCE in the breast cancer cell lines that exhibit low (SKBR3 and BT20) or high (T47D) Orai1:Orai2 expression ratios. Cells were transfected with esiOrai2 or non-specific siRNA, and the effect of Orai2 knockdown in SOCE was assessed by fluorescence microscopy. Transfection with esiOrai2 significantly attenuated the Orai2 transcript expression detected by qPCR to 44 ± 15%, 43 ± 05% and 59 ± 01% in BT20, SKBR3 and T47D cells, respectively, (*p* < 0.01; Mann–Whitney U test) and reduced the Orai2 protein expression as detected by Western blotting by about 60–70% in 48 h (Figure 2a,f,k; *p* < 0.01; Mann–Whitney U test). Furthermore, we have found that Orai2 knockdown significantly attenuated Orai2 surface expression in the cell lines investigated (Figure S2; *p* < 0.001). Additionally, we have tested whether cell transfection with esiOrai2 alters the expression of other relevant SOCE proteins. As depicted in Figure S3, Orai2 knockdown was without effect on the expression of STIM1, STIM2, Orai1 and Orai3 at the protein level. Forty-eight hours after transfection, cells were suspended in a Ca^2+^-free medium and treated with the SERCA inhibitor TG (1 µM) which resulted in an increase in the fura-2 fluorescence ratio due to passive Ca^2+^ release from the intracellular Ca^2+^ stores. Subsequent addition of Ca^2+^ to the extracellular medium resulted in a rise in the fura-2 fluorescence ratio indicative of Ca^2+^ influx (Figure 2b–d,g–i,l–n). We added increasing concentrations of extracellular Ca^2+^ (0.2–1 mM) to test the effect of Orai2 knockdown at different electrochemical Ca^2+^ gradients. As shown in Figure 2b–e, Orai2 knockdown in BT20 cells was without effect on TG-induced Ca^2+^ influx in the presence of 1 mM extracellular Ca^2+^; however, in the presence of smaller Ca^2+^ concentrations (0.3 and 0.2 mM), Orai2 expression attenuation resulted in a significantly greater SOCE (*p* < 0.05 or 0.01). It should be noted that Orai2 knockdown did not significantly modify TG-evoked Ca^2+^ release from the intracellular stores (Figure 2b–e). Similar results were observed in SKBR3 cells, which also shows a low Orai1:Orai2 expression ratio. In these cells, Orai2 knockdown significantly enhanced SOCE in the presence of 0.2 or 0.3 mM extracellular Ca^2+^ (Figure 2g–j; *p* < 0.05 or 0.01). By contrast, in T47D cells, which exhibit a high Orai1:Orai2 expression ratio, Orai2 knockdown was without effect on TG-induced Ca^2+^ influx at any Ca^2+^ concentration tested (Figure 2l–o). These findings indicate that Orai2 plays a negative modulatory role on SOCE in cells that exhibit a higher Orai2 expression.

### 2.3. Role of Orai2 in the Maintenance of Ca^2+^ Oscillations

**Figure 3:**
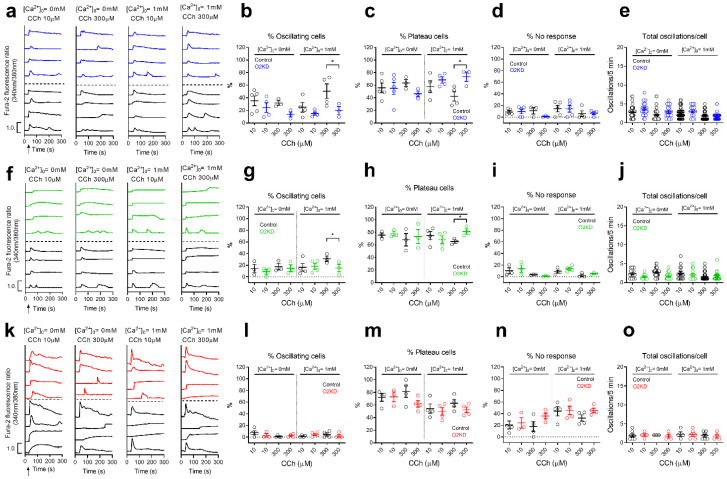
Ca^2+^ oscillations induced by CCh in breast cancer cells are not affected by Orai2 knockdown. (**a**,**f**,**k**) Representative Ca^2+^ oscillations in response to 10 or 300 µM CCh measured using Fura2 in BT20 (**a**), SKBR3 (**f**) and T47D (**k**) cells transfected with esiOrai2 (color traces) or non-specific siRNA (black traces; control). Cells were superfused with HBSS containing 1 mM Ca^2+^ and stimulated with 10 or 300 µM CCh at 1 min (indicated by arrow in the first panel). Representative traces from 4 cells/condition were chosen to represent the datasets. (**b**–**d**,**g**,**h**,**l**–**n**) Quantification of the percentage of oscillating cells (**b**,**g**,**l**), percentage of plateau cells (**c**,**h**,**m**), and percentage of non-responding cells (**d**,**i**,**n**) for data presented in (**a**,**f**,**k**) (*n*  =  3–5; n-values correspond to independent experiments). For data presented in (**e**,**j**,**o**), from left to right, *n* = 52, 25, 49, 18, 40, 15, 79 and 25 for panel (**e**), *n* = 13, 7, 28, 18, 16, 15, 49 and 18 for panel (**j**) and *n* = 10, 4, 3, 4, 3, 7, 7 and 4 for panel (**o**); *n*-values correspond to individual cells). The total number of cells analyzed in every condition is shown in Table S1. Scatter plots are represented as mean ± SEM and were statistically analyzed using ANOVA with multiple comparisons (Tukey test) to cells transfected with scramble plasmids (* *p* < 0.05).

We have further analyzed the role of Orai2 on carbachol (CCh)-induced Ca^2+^ oscillations in SKBR3, BT20 and T47D cells, as representative of breast cancer cells with low and high Orai1:Orai2 expression ratios. Cells were transfected with esiOrai2 or non-specific siRNA and were stimulated with low (10 µM) and high (300 µM) concentrations of CCh in the absence or presence of 1 mM extracellular Ca^2+^. Traces from 2–4 representative cells are shown in Figure 3a,f,k. An average of 35 and 32% of BT20 cells stimulated with CCh 10 and 300 µM, respectively, in the absence of extracellular Ca^2+^ responded with regenerative Ca^2+^ oscillations, with an average of 2.8 ± 0.2 and 2.1 ± 0.2 oscillations/5 min, respectively (Figure 3e), a pattern that was not significantly modified by Orai2 knockdown (Figure 3b,e). Most of the cells that did not oscillate responded with a sustained plateau and a low number of cells did not respond (Figure 3c,d). In the presence of extracellular Ca^2+^, an average of 28 and 55% of BT20 cells stimulated with CCh 10 and 300 µM, respectively, responded with Ca^2+^ oscillations. Orai2 knockdown significantly attenuated the percentage of cells stimulated with 300 µM CCh that responded with Ca^2+^ oscillations to 19% (Figure 3b; *p* < 0.05). Consequently, the number of cells that responded with a sustained plateau increased significantly (Figure 3c; *p* < 0.05). Similar results were found when we repeated the experiments in SKBR3, where Orai2 knockdown significantly reduced the percentage of cells stimulated with high concentration of CCh (300 µM) that responded with Ca^2+^ oscillations, thus enhancing the percentage of cells that responded with sustained plateau (Figure 3g,h; *p* < 0.05). These findings indicate that, in cells with a relatively high Orai2 expression, Orai2 plays an important role in the maintenance of regenerative Ca^2+^ oscillations. Surprisingly, T47D cells, which exhibit the highest Orai1:Orai2 expression ratio among the breast cancer cells investigated, were mostly unable to respond with Ca^2+^ oscillations in response to low and high CCh concentrations (Figure 3l), and Orai2 knockdown was unable to modify significantly the pattern of response to CCh (Figure 3l,m). Orai2 knockdown did not significantly modify the average number of oscillations per cell (Figure 3e,j,o).

### 2.4. Orai2 Modulates NFAT1 and NFAT4 Nuclear Translocation

**Figure 4:**
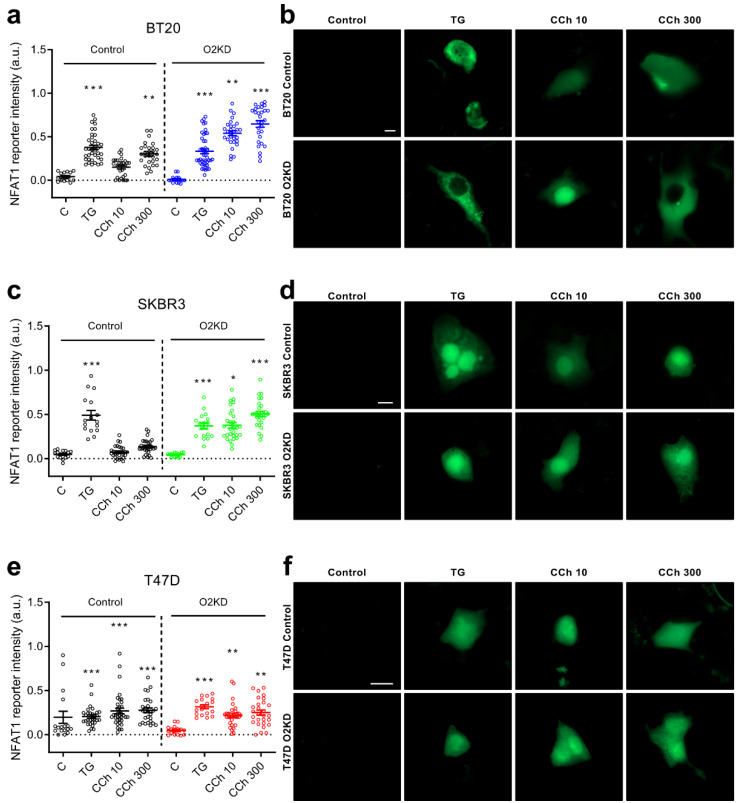
Orai2 knockdown modifies NFAT1 transcriptional activity. BT20 (**a**,**b**), SKBR3 (**c**,**d**) and T47D (**e**,**f**) cells were transfected with esiOrai2 (O2KD) or non-specific siRNA (Control), as indicated, as well as with GFP-NFAT1-reporter. Cells were stimulated with 1 µM TG or CCh (10 or 300 µM). NFAT1 reporter was determined before (Control) and 120 min after the addition of TG or CCh and fluorescence was detected using confocal microscopy, as described in Section 4. Scatter plots are represented as mean ± SEM (*n* = 15–43; *n*-values correspond to individual cells). Data were statistically analyzed using Kruskal–Wallis test with multiple comparisons (Dunn´s test) to cells transfected with scramble plasmids (* *p* < 0.05, ** *p* < 0.01 and *** *p* < 0.001). (**b**,**d**,**f**) Representative images of GFP-NFAT1-reporter in BT-20, SKBR3 and T47D cells before (Control) and 120 min after the addition of TG (1 µM) or CCh (10 or 300 µM). Scale bar: 10 µm.

SOCE has been reported as an essential regulator of NFAT nuclear translocation [27,28]. The nuclear translocation of NFAT1 has been shown to require robust SOCE activation, while NFAT4 nuclear translocation has been associated with more subtle SOCE activation [29,30]. In order to ascertain the functional role of Orai2 on NFAT1 nuclear translocation and activity, we expressed an NFAT1-responsive GFP construct (NFAT reporter), where the fluorophore is controlled by a CMV promoter and tandem repeats of the NFAT consensus binding site as previously described [31]. Cells were stimulated with TG (1 µM) or CCh (10 and 300 µM) in the presence of 1 mM extracellular Ca^2+^ for 120 min, and the abundance of GFP as indicated by the intensity of the signal was monitored. As shown in Figure 4a,b, in BT20 cells treated with non-specific siRNA, the GFP intensity was significantly enhanced by cell treatment with TG (*p* < 0.001). Furthermore, cell stimulation with CCh (300 µM) significantly enhanced GFP intensity (Figure 4a,b; *p* < 0.01), while a low concentration of CCh (10 µM) was unable to significantly enhance NFAT1 nuclear translocation (Figure 4a,b). Interestingly, in BT20 cells, where Orai2 had been knocked down, CCh was able to significantly enhance NFAT1 nuclear translocation both at 10 and 300 µM (Figure 4a,b; *p* < 0.01 or 0.001). These findings are consistent with the enhanced SOCE and the change from Ca^2+^ oscillations to more robust sustained plateau in agonist-treated cells by Orai2 knockdown, and in agreement with the previous observation that NFAT1 requires robust Ca^2+^ responses [4,28].

We repeated this experimental maneuver in SKBR3 and T47D. In SKBR3, NFAT1 nuclear translocation in response to TG enhanced GFP intensity, but treatment with high and low concentrations of CCh in the presence of 1 mM external Ca^2+^ was without effect (Figure 4c,d). Orai2 knockdown enhanced the ability of CCh to induce NFAT1 activity at the concentrations investigated (Figure 4c,d; *p* < 0.05 or 0.001). The findings observed in BT20 and SKBR3 cells are consistent with the high Orai2 expression in these cells. In T47D cells transfected with non-specific siRNA, TG and CCh enhanced NFAT1 nuclear translocation and, in contrast to BT20 and SKBR3 cells, in T47D, Orai2 knockdown did not significantly alter the ability of agonists to induce NFAT1 activation (Figure 4e,f). These findings are consistent with the low Orai2 expression in these cells and the lack of effect of Orai2 knockdown in SOCE.

**Figure 5:**
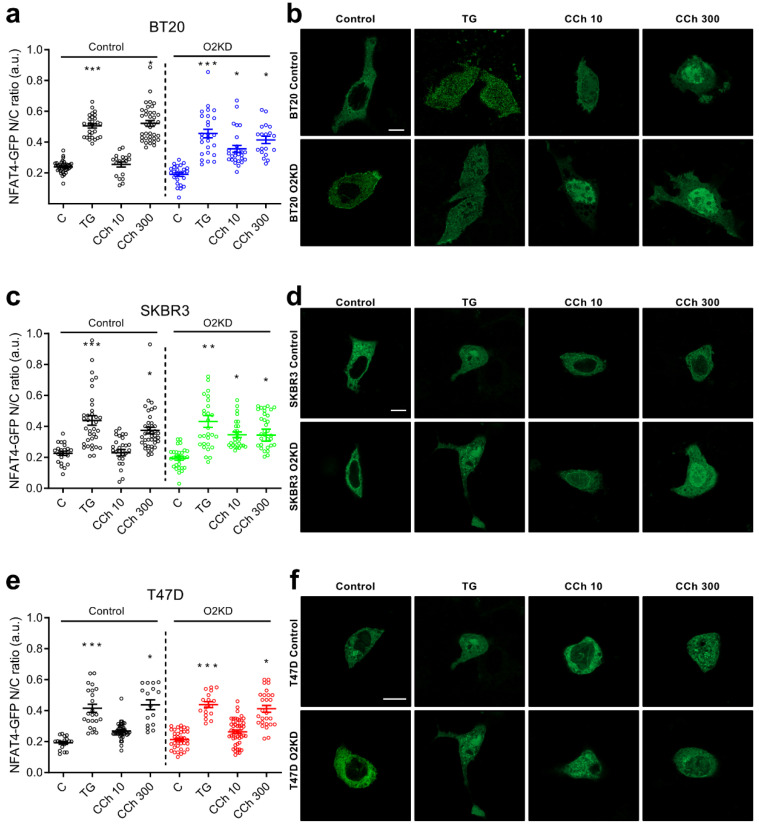
Orai2 knockdown modifies NFAT4 nuclear translocation. BT20 (**a**,**b**), SKBR3 (**c**,**d**) and T47D (**e**,**f**) cells were transfected with esiOrai2 (O2KD) or non-specific siRNA (Control), as indicated, as well as with HA-NFAT4(3-407)-GFP. Cells were stimulated with 1 µM TG or CCh (10 or 300 µM). NFAT4 nuclear translocation was determined before (Control) and 30 min after the addition of TG or CCh, and fluorescence was detected using confocal microscopy, as described in Section 4. Scatter plots represent the ratio of nuclear to total NFAT4 and is presented as mean ± SEM (*n* = 19–49; *n*-values correspond to individual cells). Data were statistically analyzed using Kruskal–Wallis test with multiple comparisons (Dunn´s test) to cells transfected with scramble plasmids (* *p* < 0.05, ** *p* < 0.01 and *** *p* < 0.001). (**b**,**d**,**f**) Representative images of NFAT4 nuclear translocation in BT-20, SKBR3 and T47D cells before (Control) and 30 min after the addition of TG (1 µM) or CCh (10 or 300 µM). Scale bar: 10 µm.

To analyze the role of Orai2 in NFAT4 nuclear translocation, we expressed the HA-NFAT4(3-407)-GFP construct and cells were stimulated with TG (1 µM) or CCh (10 and 300 µM) in the presence of 1 mM extracellular Ca^2+^ for 30 min and monitored the intensity of the GFP signal at the nuclear location. As depicted in Figure 5a,b, in BT20 cells treated with non-specific siRNA, NFAT4 nuclear translocation was significantly enhanced by cell treatment with 1 µM TG (*p* < 0.001) or 300 µM CCh (*p* < 0.05), which is consistent with the findings that NFAT4 activation requires relatively low Ca^2+^ signals [28]. Similar results were observed in SKBR3 and T47D cells (Figure 5c–f). Interestingly, Orai2 knockdown in BT20 and SKBR3 cells significantly enhances the ability of low concentrations of CCh (10 µM) to induce NFAT4 nuclear translocation (Figure 5a–d; *p* < 0.05) while, by contrast, attenuation of Orai2 expression was without a significant effect on the ability of TG or CCh to induce NFAT4 nuclear translocation in T47D cells (Figure 5e,f).

### 2.5. Orai2 Knockdown Induces Cell Cycle Arrest and Apoptosis in BT20 and SKBR3 Cells

**Figure 6:**
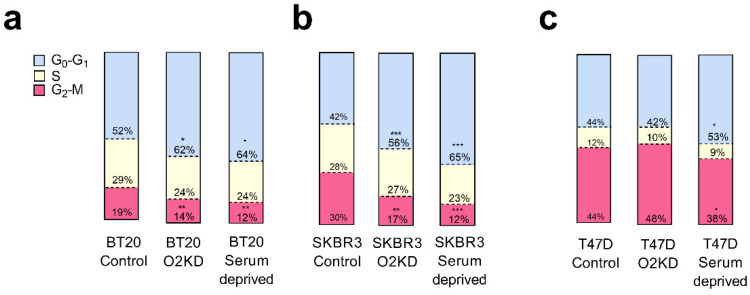
Orai2 is required for cell cycle progression. (**a**,**c**) BT20 (**a**), SKBR3 (**b**) and T47D (**c**) cells were transfected with esiOrai2 (O2KD) or non-specific siRNA (Control), as indicated, or were serum deprived. Forty-eight hours later, cell cycle analysis through PI staining and following flow cytometry was performed as described in Section 4. Stacked bars are representative of three independent experiments. Data were statistically analyzed using Kruskal–Wallis test with multiple comparisons (Dunn´s test) to cells transfected with non-specific siRNA (* *p* < 0.05, ** *p* < 0.01 and *** *p* < 0.001).

Next, we investigated whether Orai2 plays a relevant role in cell cycle progression and apoptosis resistance in BT20 and SKBR3 cells, which exhibit the greater Orai2 expression among the breast cancer cell lines investigated, and also in T47D, which shows a low Orai2 expression. To investigate this issue, we transfected BT20, SKBR3 and T47D cells with esiOrai2 or non-specific siRNA, as control, and analyzed cell cycle parameters. Cells serum deprived for 24 h were used as positive control (Figure 6a–c). We found that BT20 and SKBR3 cells transfected with esiOrai2 displayed a G1 arrest (Figure 6a,b; *p* < 0.05 and 0.001 for BT20 and SKBR3, respectively, and Figure S6). Similarly, serum deprivation resulted in G1 arrest in all the cells investigated (Figure 6a–c; *p* < 0.05 for BT20 and T47D and *p* < 0.001 for SKBR3). These findings indicate a functional role of Orai2 in cell cycle progression in BT20 and SKBR3 cells.

**Figure 7:**
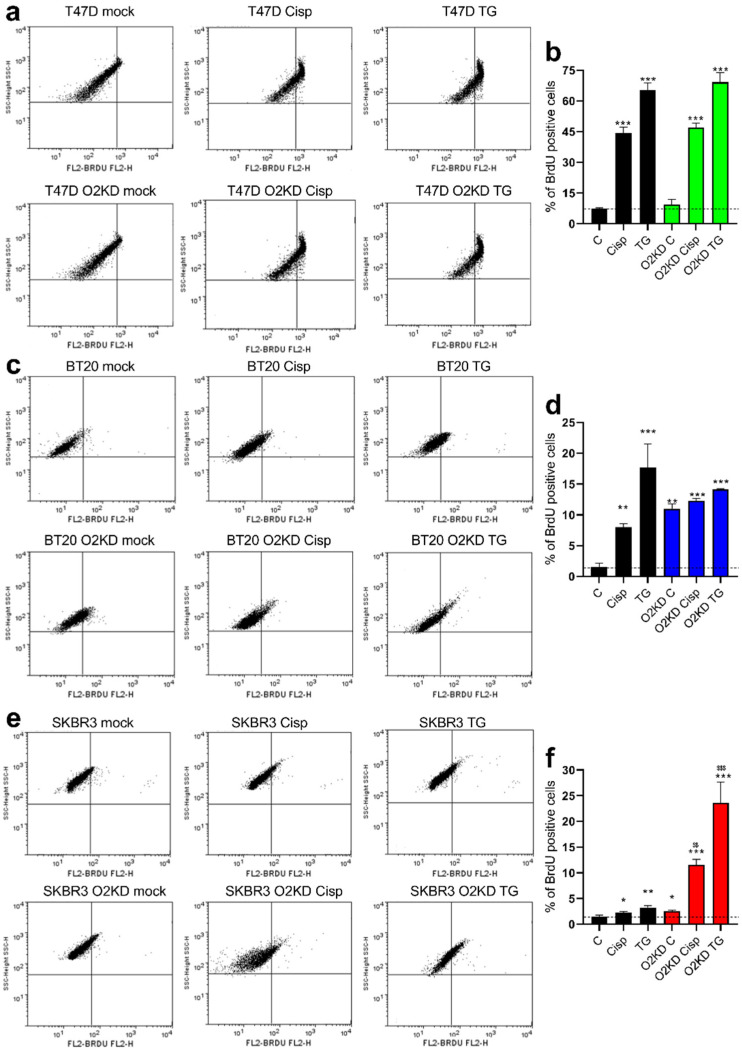
Orai2 is required for apoptosis resistance. T47D (**a**,**b**), BT20 (**c**,**d**) and SKBR3 (**e**,**f**) cells were transfected with esiOrai2 (O2KD) or non-specific siRNAscramble plasmids, as indicated. Forty-eight hours later cells were treated with cisplatin (50 µM) for 30 min or TG (2 µM) for 2 h, and the percentage of apoptotic cells was assessed using the in situ BrdU-Red DNA Fragmentation (TUNEL) commercial kit, as described in Section 4. Bar graphs are representative of three different experiments expressed as mean ± S.E.M. *, **, ***: represent *p* < 0.05, *p* < 0.01 and *p* < 0.001 as compared to cells transfected with non-specific siRNA scramble plasmids and not treated with cisplatin or TG. $$, $$$: represent *p* < 0.01 and *p* < 0.001 as compared to cells transfected with esiOrai2 but not treated with cisplatin or TG.

We further analyzed the role of Orai2 in apoptosis resistance in T47D, BT20 and SKBR3 cells by flow-cytometry and using the BrdU-based TUNEL assay. As depicted in Figure 7, in cells treated with non-specific siRNA, treatment with 50 µM cisplatin or 1 µM TG significantly enhanced percentages of BrdU-positive cells (for T47D cells *p* < 0.001 for cisplatin and TG; for BT20 cells *p* < 0.01 and 0.001 for cisplatin and TG, respectively; for SKBR3 cells *p* < 0.05 and 0.01 for cisplatin and TG, respectively). The effect of cisplatin and TG on the development of apoptosis was especially remarkable in T47D and BT20 (Figure 7b,d vs. Figure 7f). Orai2 knockdown had no significant effect on the percentage of apoptotic T47D cells (Figure 7a,b). By contrast, Orai2 knockdown enhanced per se the percentage of apoptotic BT20 and SKBR3 cells and significantly increased the percentage of apoptotic cells upon treatment with cisplatin (Figure 7d,f; *p* < 0.001). These findings indicate that Orai2 plays a relevant role in apoptosis resistance in cells with a high expression of this channel.

## 3. Discussion

Breast cancer is a heterogeneous disorder that shows great diversity between and within tumor subtypes, and this heterogeneity determines cancer cell biology and, subsequently, chemoresistance and prognosis. Orai1 is the essential component of the CRAC channels [32,33], but Orai2 and Orai3 have been reported to modulate CRAC channel activity to match Ca^2+^ signals to agonist stimulation [30]. Here we provide evidence of the heterogeneous expression of Orai1 and Orai2 between different breast cancer cell lines which leads to the identification of breast cancer cells with high and low Orai1:Orai2 expression ratios. In neoplastic cell lines with a high Orai2 expression, as compared to pre-neoplastic MCF10A cells, we have found that Orai2 negatively modulates TG-induced SOCE and plays an important role in the maintenance of regenerative Ca^2+^ oscillations. The later are transient rises in cytosolic Ca^2+^ concentration that arise from the regenerative discharge of stored Ca^2+^ upon cell stimulation with modest, physiological concentrations of calcium-mobilizing agonists [34], while greater agonist concentrations lead to more sustained Ca^2+^ signals [35]. Consistent with this, our results indicate that Orai2 knockdown leads to more extensive Ca^2+^ influx, which then switches the agonist-mediated Ca^2+^ responses from Ca^2+^ oscillations to Ca^2+^ plateaus.

As a result of the increase in agonist-induced Ca^2+^ responses upon Orai2 knockdown, attenuation of Orai2 expression enhanced the efficacy of agonists to increased nuclear translocation of NFAT1 and NFAT4. NFAT1 and NFAT4 are two isoforms of the family of nuclear factors of activated T cells that are expressed in a variety of cells and tissues where they are involved in the transcription of a variety of genes [36]. NFAT1-4 are predominantly located in the cytosol and their activities are controlled by the Ca^2+^/calmodulin-dependent serine/threonine phosphatase, calcineurin [37]. NFAT1 and NFAT4 activation requires Ca^2+^ influx, but unlike NFAT1, which requires robust SOCE activation, NFAT4 activation has been associated to more subtle SOCE activation [29,30]. Our results indicate that in BT20 and SKBR3 cell types, which exhibit high endogenous expression of Orai2, stimulation with a low concentration (10 µM) of the cholinergic agonist CCh is unable to induce NFAT1 activation, and a high CCh concentration (300 µM) was unable to induce NFAT1 nuclear translocation in SKBR3 cells. By contrast, in T47D, showing a high Orai1:Orai2 expression ratio, CCh was able to induce NFAT1 activation even at low agonist concentration. Interestingly, Orai2 knockdown in BT20 and SKBR3 cells enhances the ability of CCh to induce NFAT1 activation. A recent study has reported that Orai1, but not Orai2 or Orai3, is able to interact with the scaffolding protein AKAP79 (A-kinase anchoring protein 79) [38], which facilitates the interaction between NFAT and calcineurin [39]. Therefore, the role of Orai2 in the regulation of NFAT nuclear translocation should be attributed to the modulation of the magnitude of Ca^2+^ entry via Orai1/CRAC channels rather than to a direct effect on NFAT dephosphorylation. Similarly, while cell stimulation with a low agonist concentration was unable to induce nuclear translocation of NFAT4 in all the cells investigated, in BT20 and SKBR3 cells transfected with esiOrai2, stimulation with a low concentration of CCh was able to induce NFAT4 nuclear translocation. By contrast, as expected, in cells with a low endogenous Orai2 expression, such as T47D cells, Orai2 knockdown did not result in a modification in the Ca^2+^ responses to agonists or the activation of NFAT1 or NFAT4. These findings indicate that in cells with a low Orai1:Orai2 expression ratio, Orai2 modulates agonist-evoked Ca^2+^ responses, thus regulating the activation of NFAT1 and NFAT4.

Our findings are consistent with the regulatory role of Orai2, as well as Orai3, in SOCE previously reported in different cell types [4,5,6,7,8,9]. According to this, Orai2 and Orai3, heteromerize with Orai1 channels and play an essential role in mediating the regenerative Ca^2+^ oscillations induced by physiological receptor occupation, thus leading to graded Ca^2+^ signals that match the strength of agonist stimulation to fine-tune transcriptional control via NFAT1 and NFAT4 [4]. Thus, our results indicate that in cells with a high Orai2 expression (BT20 and SKBR3) NFAT1 and NFAT4 nuclear translocation discriminate between low and high concentrations of agonist (except for NFAT1 activation in SKBR3), whereas in BT20 and SKBR3 cells where Orai2 expression has been silenced NFAT1 and NFAT4 activation barely discriminate between high and low agonist concentrations.

In cells with a relevant expression of Orai2, such as BT20 and SKBR3 cells, Orai2 knockdown resulted in cell cycle arrest at G1. This process might underlie the increase in the development of apoptotic events in cells where Orai2 expression had been attenuated. Furthermore, our findings reveal that BT20 and SKBR3 cells are more susceptible to cisplatin treatment upon Orai2 knockdown, thus indicating that Orai2 confers apoptosis resistance to the cells investigated.

## 4. Materials and Methods

### 4.1. Materials and Reagents

Fura-2 acetoxymethyl ester (fura-2/AM) was from Molecular Probes (Leiden, The Netherlands). High-glucose Dulbecco’s modified Eagle’s medium (DMEM), fetal bovine serum, trypsin, penicillin/streptomycin, Clean-Blot™ IP detection reagent, SuperSignal^®^ West Dura extended duration substrate reagent and Pierce™ BCA protein assay kits were purchased from ThermoFisher Scientific (Waltham, MA, USA). Complete EDTA-free protease inhibitor cocktail tablets were from Roche Diagnostics GmbH (Mannheim, Germany). DharmaFECT kb transfection reagent was obtained from Horizon Discovery (Waterbeach, UK). GFP-NFAT1-reporter overexpression plasmid was kindly provided by Christoph Romanin (Johannes Kepler Institute of Biophysic, University of Linz, Linz, Austria). HA-NFAT4(3-407)-GFP was a gift from Anjana Rao (Addgene plasmid # 21664; http://n2t.net/addgene:21664, accessed on 20 November 2021; RRID:Addgene_21664). Propidium iodide (PI) was from Immunostep (Salamanca, Spain). Thapsigargin (TG), carbachol (CCh), HEPES (4-(2-Hydroxyethyl)piperazine-1-ethanesulfonic acid), esiRNA Orai2 (a heterogeneous mixture of siRNA that all target the same mRNA sequence), EGTA (ethylene glycol-bis(2-aminoethylether)-*N*,*N*,*N*′,*N*′-tetraacetic acid), EDTA (ethylenedinitrilotetraacetic acid), bovine serum albumin (BSA), sodium azide, rabbit polyclonal anti-Orai1 antibody (catalog number O8264, epitope: amino acids 288-301 of human Orai1) and rabbit polyclonal anti-β-actin antibody (catalog number A2066, epitope: amino acids 365-375 of human β-actin) were obtained from Sigma (St Louis, MO, USA). Rabbit polyclonal anti-Orai2 antibody (catalog number TA306419, epitope: sequence localized in the C-terminal region) was from Acris (Herford, Germany). Horseradish peroxidase-conjugated goat anti-mouse immunoglobulin G (IgG) antibody (catalog number 115-035-003) and goat anti-rabbit IgG antibody (catalog number 111-035-003) were from Jackson Laboratories (West Grove, PA, USA). SiRNA-A was purchased from Santa Cruz Biotechnology (Heidelberg, Germany). All other reagents were of an analytical grade.

### 4.2. Cell Culture and Transfections

The MCF10A cell line was provided by Dr. Potier-Cartereau (Université François Rabelais Tours, France). SKBR3, T47D, MCF7, BT-20, MDA-MB-468 and MDA-MB-231 cell lines were obtained from American Type Culture Collection (ATCC, Manassas, VA, USA). Cells were cultured up to 20–25 passages at 37 °C with 5% CO_2_ in Dulbecco’s Modified Eagle Medium (DMEM) or DMEM-F12 (MCF10A), supplemented with 10% (*v*/*v*) fetal bovine serum (or fetal horse serum for MCF10A) and 100 U/mL penicillin and streptomycin, as described previously [20]. For transient transfections, cells were grown to 60–80% confluency and transfected with esiOrai2 or non-specific siRNA using DharmaFECT kb transfection reagent and were used 48 h after transfection. For Western blotting assays, cells (2 × 10^6^) were plated in 75-cm^2^ flasks and cultured for 48–72 h, while, for calcium imaging, NFAT assays and cytometry, cells (2 × 10^5^–4 × 10^5^) were seeded in a 35-mm six-well multidish.

### 4.3. RNA Extraction and qRT-PCR

RNAs were extracted from MCF10A breast epithelial cells and breast cancer cells using TRIzol^®^ reagent (Invitrogen, Carlsbad, CA, USA) according to the manufacturer’s specifications. The primers used are: hOrai1 (forward primer: AGCAACGTGCACAATCTCAA; reverse primer: GTCTTATGGCTAACCAGTGA), hOrai2 (forward primer: CGGCCATAAGGGCATGGATT; reverse primer: TTGTGGATGTTGCTCACGGC) and GAPDH (forward primer: CTAGGCGCTCACTGTTCTCTC; reverse primer: GTCCGAGCGCTGACCTT). SYBR green qRT-PCR was performed using SYBR^®^ Premix Ex Taq™ (Takara Bio Inc., Otsu, Shiga, Japan) in an Applied Biosystems STEPONE Real-Time thermal cycler (Life Technologies Corporation, Carlsbad, CA, USA) as described previously [40]. PCR products were obtained using the following cycling conditions: 96 °C for 2 min, followed by 35 cycles of 96 °C for 15 s, 48–56 °C for 25 s and finished with 72 °C for 10 min. mRNA abundance was calculated by the comparative CT (ΔΔCT) method using the formula RQ = 2^−ΔΔCT^. The amount of mRNA transcripts was normalized to GAPDH expression and represented as mean expression ± S.E.M.

### 4.4. Western Blotting

Western blotting was performed as described previously [41]. Briefly, MCF10A, SKBR3, T47D, MCF7, BT-20, MDA-MB-468 and MDA-MB-231 cell lines were harvested and lysed with ice-cold 2×Nonidet P-40 buffer, pH 8, containing 274 mM NaCl, 40 mM Tris, 4 mM EDTA, 20% glycerol, 2% Nonidet P-40, 2 mM Na_3_VO_4_ and complete EDTA-free protease inhibitor tablets. Separated proteins were electrophoretically transferred onto nitrocellulose membranes for subsequent probing. Blots were incubated overnight with 10% (*w*/*v*) BSA in Tris-buffered saline with 0.1% Tween-20 (TBST) to block residual protein binding sites. Immunodetection of Orai1, Orai2 and β-actin was achieved by incubation for 2 h with anti-Orai1 or anti-Orai2 antibody diluted 1:1000 in TBST and 1 h with anti-β-actin antibody diluted 1:2000 in TBST, respectively. The primary antibody was removed, and blots were washed three times for 10 min each with TBST. To detect the primary antibody, blots were incubated for 1 h with horseradish peroxidase-conjugated goat anti-rabbit or anti-mouse IgG antibody diluted 1:10,000 in TBST and then exposed to enhanced chemiluminescence reagents for 5 min. The density of bands was recorded using a C-DiGit Chemiluminescent Western Blot Scanner (LI-COR Biosciences, Lincoln, NE, USA) and measured using Fiji ImageJ software v.153f51 (NIH, Bethesda, MD, USA). Data were normalized to β-actin from the same gel.

### 4.5. Determination of Cytosolic Free-Calcium Concentration ([Ca^2+^]_c_)

Cells were loaded with fura-2 by incubation with 2 μM fura-2/AM for 30 min at 37 °C as described previously [42]. Coverslips with cultured cells were mounted on a perfusion chamber and placed on the stage of an epifluorescence inverted microscope (Nikon Eclipse Ti2, Amsterdam, The Netherlands) with an image acquisition and analysis system for video microscopy (NIS-Elements Imaging Software v.5.02.00, Nikon, Amsterdam, The Netherlands). Cells were continuously superfused at room temperature with HEPES-buffered saline (HBS) containing (in mM) 125 NaCl, 5 KCl, 1 MgCl2, 5 glucose, and 25 HEPES, pH 7.4, supplemented with 0.1% (*w*/*v*) BSA. Cells were examined at 40× magnification (Nikon CFI S FLUOR 40× Oil, Amsterdam, The Netherlands) and were alternatively excited with light from a xenon lamp passed through a high-speed monochromator Optoscan ELE 450 (Cairn Research; Faversham, UK) at 340/380 nm. Fluorescence emission at 505 nm was detected using a cooled digital sCMOS camera Zyla 4.2 (Andor; Belfast, UK) and recorded using NIS-Elements AR software (Nikon; Tokyo, Japan). Fluorescence ratio (F340/F380) was calculated pixel by pixel, and the data were presented as ΔF_340_/F_380_, as previously described [43]. TG-evoked Ca^2+^ release and Ca^2+^ mobilization, as well as SOCE in cells were estimated as the area under the curve (AUC) measured as the integral of the rise in fura-2 fluorescence ratio for 2 min after the addition of TG in the absence or presence of extracellular Ca^2+^, respectively, taking a sample every second. To compare the rate of increase in fura-2 fluorescence between different treatments we used the constant of the exponential increase. Traces were fitted to the equation y = A(1−e^−K^^1T^) × e^−K^^2T^, where K_1_ is the constant of the exponential increase. Ca^2+^ oscillations were analyzed as described previously [4].

### 4.6. NFAT4 Nuclear Translocation Assay

For NFAT4 nuclear translocation, SKBR3, T47D and BT-20 breast cancer cells were transfected with 4 µg of HA-NFAT4(3-407)-GFP using DharmaFECT kb transfection reagent. Nuclei were stained by using DAPI (1:5000). Cells were imaged 48 h post transfection using a confocal microscope (LSM900, Zeiss, Oberkochen, Germany) with an image acquisition and analysis system for video microscopy (ZEN Software, Zeiss, Oberkochen, Germany) 40× water immersion objective. After baseline recordings, thapsigargin (TG; 1 µM) or carbachol (CCh; 10 or 300 µM) were added. Using Fiji ImageJ software v.153f51 (NIH, Bethesda, MD, USA), ROIs from DAPI images (blue), corresponding to nuclei (based on DAPI counterstaining) were superimposed upon the NFAT image. Then, mean fluorescence intensity was measured for each nuclear area and each cytoplasmic area. ROIs for each cell were delineated with the Otsu threshold algorithm, and the ratio of nuclear to total NFAT was calculated on a cell-by-cell basis.

### 4.7. NFAT1-Driven GFP Expression

BT20, SKBR3 and T47D cells were transfected with 5 µg of GFP-NFAT1-reporter. Forty-eight hours after transfection, cells were treated with 1 µM thapsigargin or CCh (10 or 300 µM) for 120 min in a 1 mM Ca^2+^−containing medium. Cells expressing NFAT-driven GFP were monitored using an Axio Observer 7 epifluorescence system (Zeiss, Oberkochen, Germany) with an image acquisition and analysis system for videomicroscopy (ZEN Software, Zeiss, Oberkochen, Germany) 25× water immersion objective and an Axiocam 712 mono, where fluorescence was recorded from individual cells with excitation of 488 nm. Cellular GFP fluorescence intensity was calculated using Fiji ImageJ software v.153f51 (NIH, Bethesda, MD, USA). ROIs corresponding to cells were selected and then superimposed upon the GFP image. Then, GFP fluorescence intensity was measured for each ROI, delineated with the Otsu threshold algorithm.

### 4.8. Determination of Cell Cycle by Flow Cytometry

Cell cycle was evaluated using propidium iodide (PI). Forty-eight hours after transfection or 24 h after serum deprivation, 2 × 10^6^ cells were fixed with EtOH 70% at −20 °C for 12–24 h. Next, EtOH was removed and washed twice with fresh ice-cold PBS and the cells were permeabilized with PBS containing 0.1% (*v*/*v*) Triton X-100 and 100 µg/mL RNase at 37 °C for 15–30 min. The percentages of cell cycle stages were defined by incubating propidium iodide (2 µM) at 37 °C for 30 min in darkness following manufacturer´s instructions. To analyze cell cycle status, cellular DNA content was estimated using a FACS-SCAN flow cytometer (Becton Dickinson, Madrid, Spain). Forward and side scattering were considered to select appropriated cells. The fluorescence emitted from cells was acquired at a wavelength of 555/624 nm (Ex/Em) for PI.

The percentage of SKBR3 and BT-20 apoptotic cells was estimated using in situ BrdU-Red DNA Fragmentation (TUNEL) commercial kit from Abcam^®^ (Cambridge, UK) as described previously [44]. Briefly, SKBR3 and BT-20 cells were grown until appropriated confluence and treated with the antineoplastic drug cisplatin (50 µM) or TG (2 µM) for 30 min. Then, the amount of BrdU incorporated into the cells was developed using an anti-BrdU-red antibody. Finally, cells were analyzed using a FACS-SCAN flow cytometer (Becton Dickinson, Madrid, Spain). Forward and side scattering were considered to select appropriated cells. The fluorescence emitted from cells was acquired at a wavelength of 488/576 nm (Ex/Em) for BrdU. The percentage of apoptotic cells was estimated by comparing the medians obtained from the different cell populations upon analyzing the dot-plot graphs using the Flowing free-software, available from the Cell Imaging Core, Turku Centre for Biotechnology, at the University of Turku and Åbo Akademi University (http://flowingsoftware.btk.fi, accessed on 20 November 2021). Data are presented as the mean percentage ± S.E.M. of positive BrdU stained cells as a result of the different treatments.

### 4.9. Statistical Analysis

All data are presented as the mean ± standard error of mean (SEM). Analysis of statistical significance was performed using GraphPad Prism v.8.4.3 (GraphPad Software, San Diego, CA, USA). A Kruskal–Wallis test combined with a Dunn´s post hoc test (or one-way analysis of variance combined with Tukey post hoc test for the analysis of Ca^2+^ determinations) were used to compare the different experimental groups. For comparison between two groups, the Mann–Whitney U test (or Student´s *t* test for the analysis of Ca^2+^ determinations) was used. Throughout the manuscript *, **, and *** indicate *p*-values of <0.05, <0.01, and <0.001, respectively. All data with *p*  <  0.05 was deemed significant; “ns”  =  non-significant.

## 5. Conclusions

In Summary, our results reveal the great heterogeneity in Orai1 and Orai2 channel expression between different breast cancer cell lines. In cells with a high Orai2 expression (low Orai1:Orai2 expression ratio) Orai2 plays a relevant role modulating SOCE and agonist-induced Ca^2+^ responses, which, in turn, leads to the modulation of NFAT1 and NFAT4 nuclear translocation. Finally, Orai2 plays an important role in cell cycle progression, which might underlie chemoresistance in breast cancer cells.

## Data Availability

The data presented in this study are available on request from the corresponding author.

## Supplementary Materials

The following are available online at https://www.mdpi.com/article/10.3390/cancers14010114/s1, Figure S1: Orai1 and Orai2 transcript expression in patients with breast cancer, stratified based on cancer subtypes (UALCAN analysis). Figure S2: Effect of Orai2 knockdown on Orai2 surface expression in T47D, SKBR3 and BT20 breast cancer cells. Figure S3: Effect of Orai2 knockdown on the expression of STIM1, STIM2, Orai1 and Orai3 in T47D, SKBR3 and BT20 breast cancer cells. Figure S4: Orai2 knockdown modifies NFAT1 transcriptional activity. Figure S5: Orai2 knockdown modifies NFAT4 nuclear translocation. Figure S6: Histogram of DNA content (propidium iodide, PI) versus number of cell counts (cell number), obtained by flow cytometry. Figure S7: Uncropped Western blots of Figure 1a,b and Figure 2a,f,k, Figures S2 and S3, Table S1: Total number of cells analyzed in Figure 3.
