# Correction: Sanchez-Collado et al. Orai2 Modulates Store-Operated Ca^2+^ Entry and Cell Cycle Progression in Breast Cancer Cells. *Cancers* 2021, *14*, 114

**DOI:** 10.3390/cancers15041290

**Published:** 2023-02-17

**Authors:** Jose Sanchez-Collado, Jose J. Lopez, Carlos Cantonero, Isaac Jardin, Sergio Regodón, Pedro C. Redondo, Juan Gordillo, Tarik Smani, Gines M. Salido, Juan A. Rosado

**Affiliations:** 1 Cellular Physiology Research Group, Department of Physiology, Institute of Molecular Pathology Biomarkers (IMPB), University of Extremadura, 10003 Caceres, Spain; 2 Pathology Service, Hospital de Merida, 06800 Merida, Spain; 3 Department of Medical Physiology and Biophysics, University of Seville, 41004 Seville, Spain; 4 Group of Cardiovascular Pathophysiology, Institute of Biomedicine of Seville, University Hospital of Virgen del Rocio, University of Seville, CSIC, 41004 Seville, Spain

## Error in Figure

In the original publication [1], there was a mistake in **Figure 5, panes d and f** as published. **We have noticed that, in panels d and f, the images of TG in SKBR3 cells (d) have been unintentionally duplicated in panel f (T47D cells).** The corrected **Figure 5** appears below.




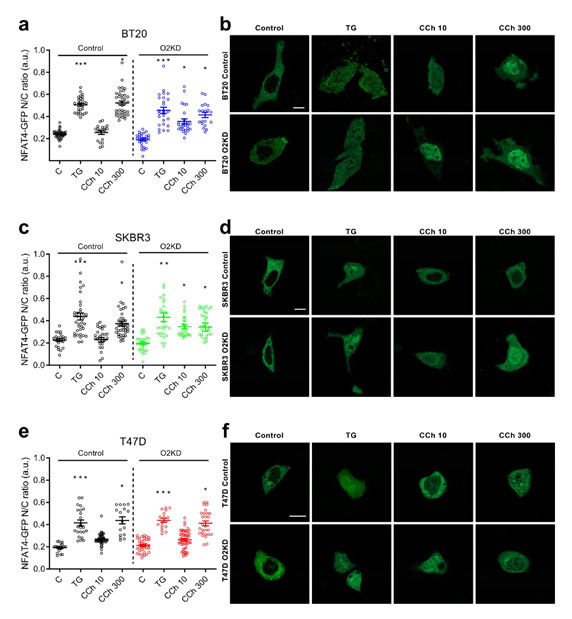




The authors apologize for any inconvenience caused and state that the scientific conclusions are unaffected. This correction was approved by the Academic Editor. The original publication has also been updated.
