# Supplementary material for: Orai2 Modulates Store-Operated Ca2+ Entry and Cell Cycle Progression in Breast Cancer Cells
Source: Cancers (Basel). 2021 Dec 27;14(1):114. doi: 10.3390/cancers14010114 (PMC8749845; doi:10.3390/cancers14010114)
Supplement: Supplementary file 1 [file cancers-14-00114-s001.zip › cancers-1503193-supplementary.pdf]

Supplementary Materials

Orai2 modulates store-operated Ca^{2+} entry and cell cycle progression in breast cancer cells

Jose Sanchez-Collado, Jose J. Lopez, Carlos Cantonero, Isaac Jardin, Sergio Regodón, Pedro C. Redondo, Juan Gordillo, Tarik Smani, Gines M. Salido and Juan A. Rosado*

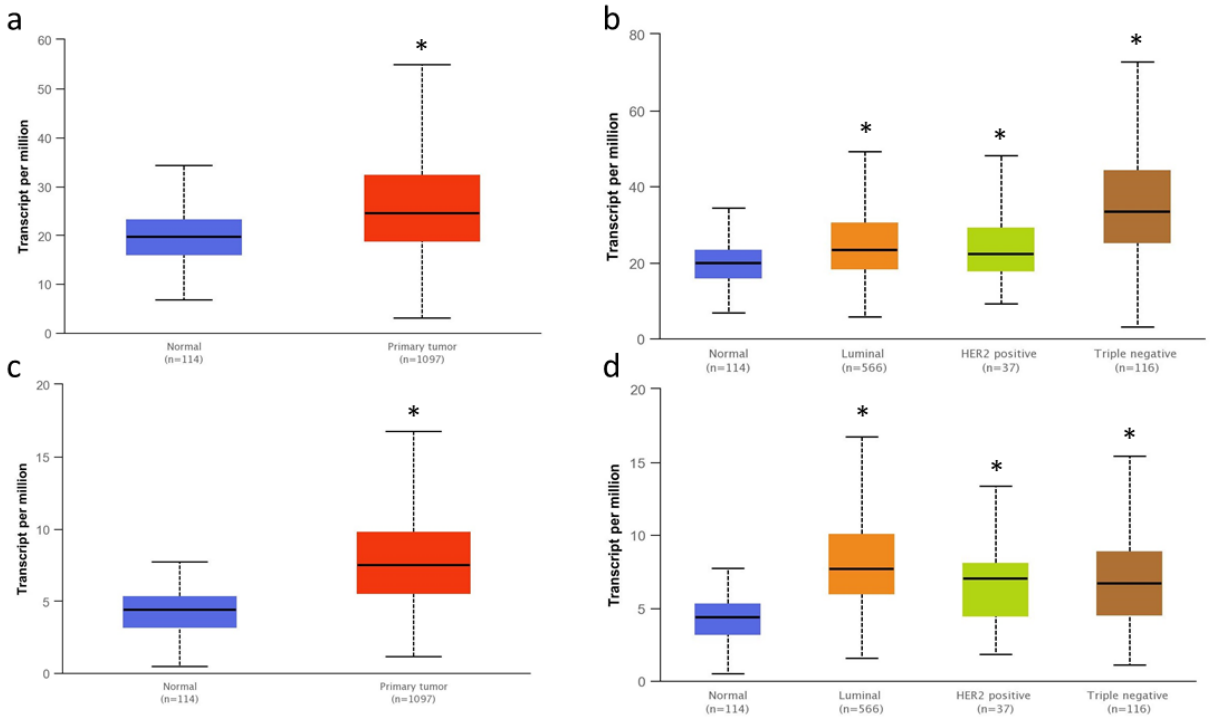

Figure S1. Orai1 and Orai2 transcript expression in patients with breast cancer, stratified based on cancer subtypes (UALCAN analysis). (**a** and **c**) Box plot showing relative expression of Orai1 (**a**) and Orai2 (**c**) in normal and breast cancer samples. (**b** and **d**) Box plots comparing the relative expression of Orai1 (**b**) and Orai2 (**d**) in normal subjects and breast cancer patients stratified by cancer subtypes. Data represents the minimum, first quartile, median, third quartile, and maximum. * $p < 0.001$.

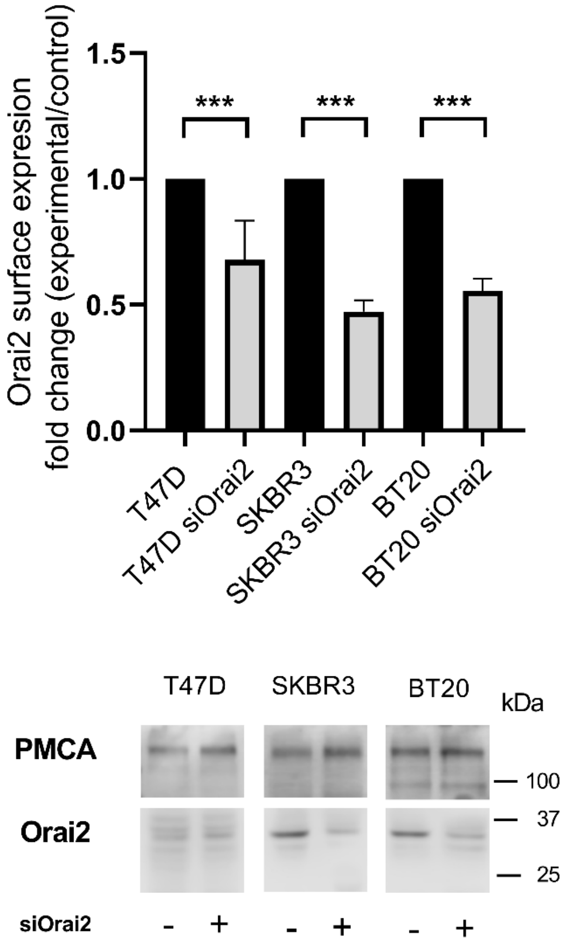

Figure S2. Effect of Orai2 knockdown on Orai2 surface expression in T47D, SKBR3 and BT20 breast cancer cells. T47D, SKBR3 and BT20 cells were transfected with esiOrai2 (siOrai2) or non-specific siRNA as control. Forty-eight hours after transfection, plasma membrane resident proteins were labeled by biotinylation, as described under Material and Methods. The biotinylated fraction was separated in 10% SDS-PAGE and analyzed by Western blotting using anti-Orai2 antibody. Membranes were reprobed with anti-PMCA antibody, as control. Positions of molecular mass markers are shown on the right. These results are representative of four separate experiments. Bar graphs represent the quantification of Orai2 surface exposition. Results are recorded as arbitrary optical density units, expressed as mean ± S.E.M. and presented as fold change (experimental/control). *** $p < 0.001$ as compared to cells transfected with non-specific siRNA.

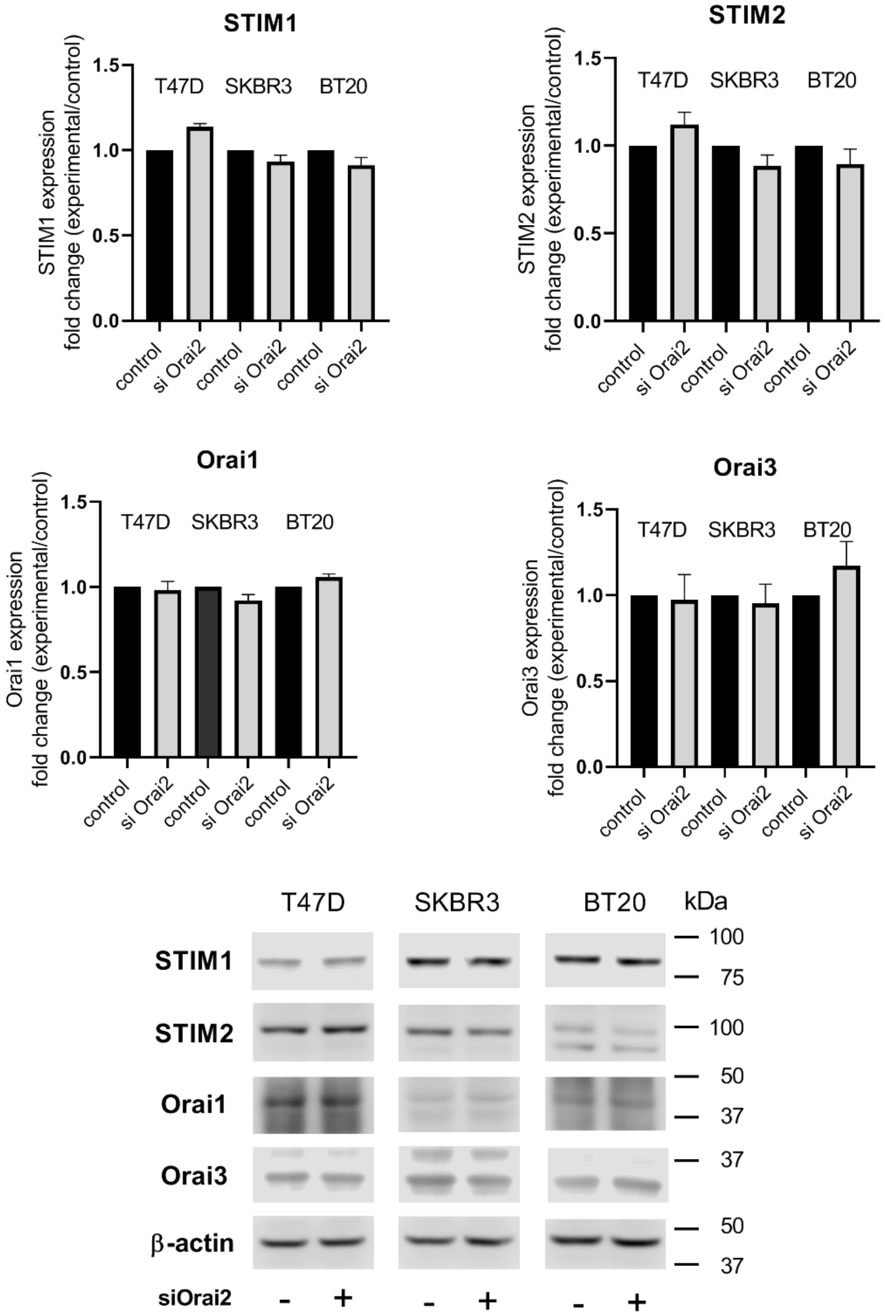

Figure S3. Effect of Orai2 knockdown on the expression of STIM1, STIM2, Orai1 and Orai3 in T47D, SKBR3 and BT20 breast cancer cells. T47D, SKBR3 and BT20 cells were transfected with esiOrai2 (siOrai2) or non-specific siRNA as control. Forty-eight hours after transfection, cells were lysed and whole cell lysates were separated in 10% SDS-PAGE and analyzed by Western blotting using anti-STIM1, anti-STIM2, anti-Orai1 or anti-Orai3 antibody. Membranes were reprobed with anti-β-actin antibody, for protein loading control. Positions of molecular mass markers are shown on the right. These results are representative of three separate experiments. Bar graphs represent the quantification of STIM1, STIM2, Orai1 or Orai3 protein expression. Results are recorded as arbitrary optical density units, expressed as mean ± S.E.M. and presented as fold change (experimental/control).

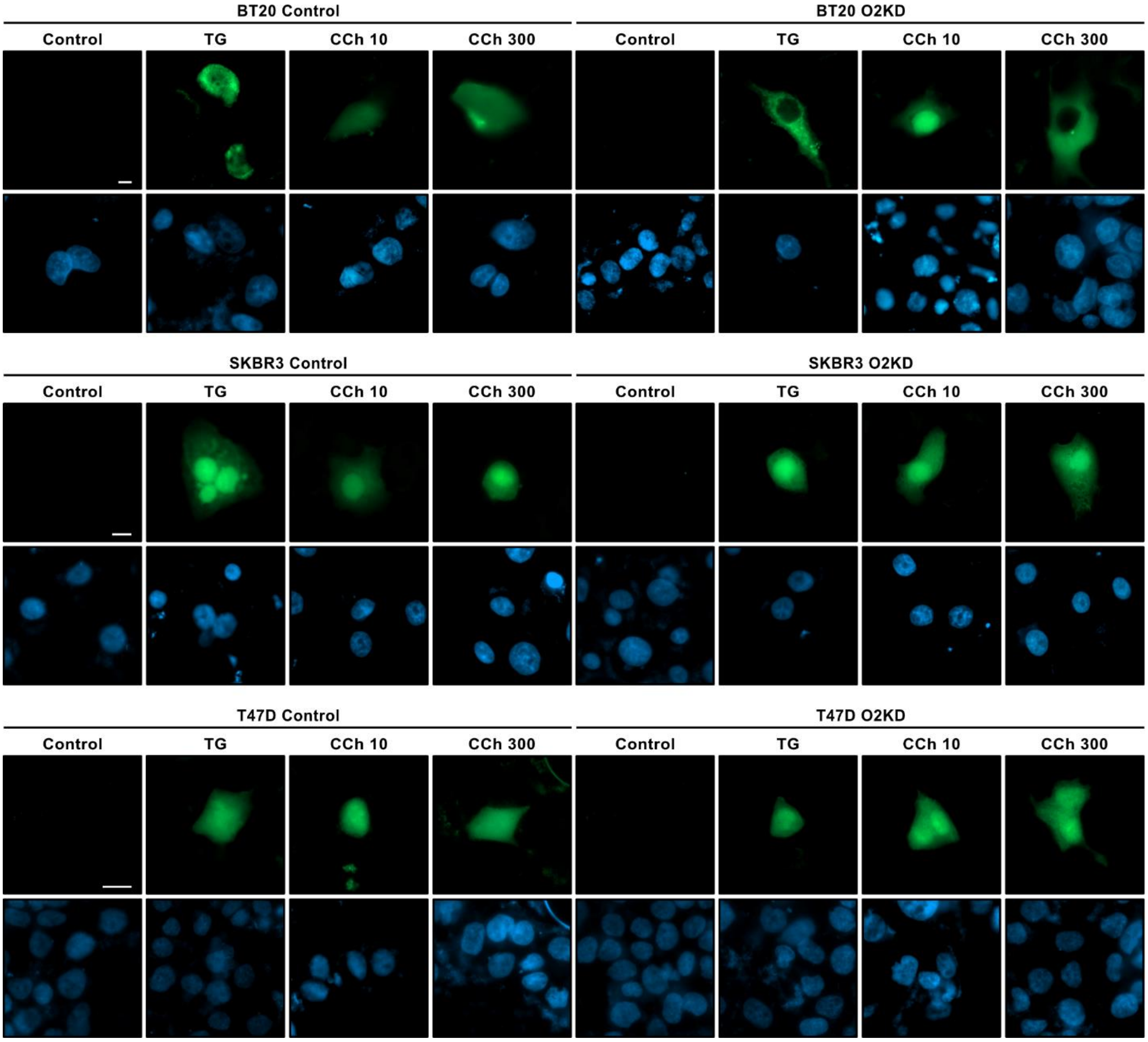

Figure S4. Orai2 knockdown modifies NFAT1 transcriptional activity. BT20, SKBR3 and T47D cells were transfected with esiOrai2 (O2KD) or non-specific siRNA (Control), as indicated, as well as with GFP-NFAT1-reporter. Cells were stimulated with 1 μM TG or CCh (10 or 300 μM). NFAT1 reporter was determined before (Control) and 120 min after the addition of TG or CCh and fluorescence was detected using an epifluorescence inverted microscope, as described in "Materials and Methods". Representative images of GFP-NFAT1-reporter and DAPI nuclear staining. Scale bar: 10 μm.

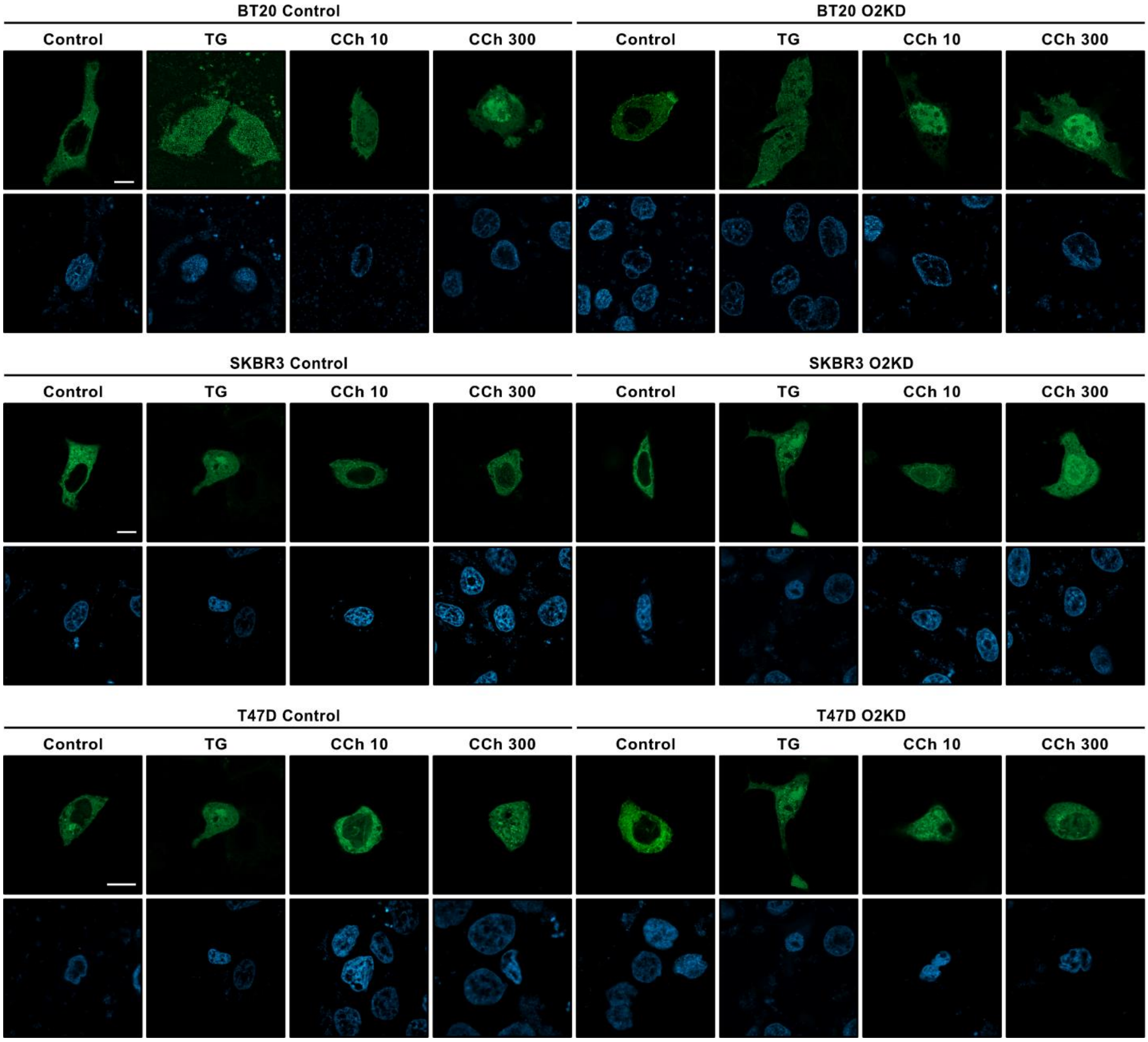

Figure S5. Orai2 knockdown modifies NFAT4 nuclear translocation. BT20, SKBR3 and T47D cells were transfected with esiOrai2 (O2KD) or non-specific siRNA (Control), as indicated, as well as with HA-NFAT4(3-407)-GFP. Cells were stimulated with 1 μM TG or CCh (10 or 300 μM). NFAT4 nuclear translocation was determined before (Control) and 30 min after the addition of TG or CCh and fluorescence was detected using confocal microscopy, as described in "Materials and Methods". Representative images of NFAT4 nuclear translocation and DAPI nuclear staining. Scale bar: 10 μm.

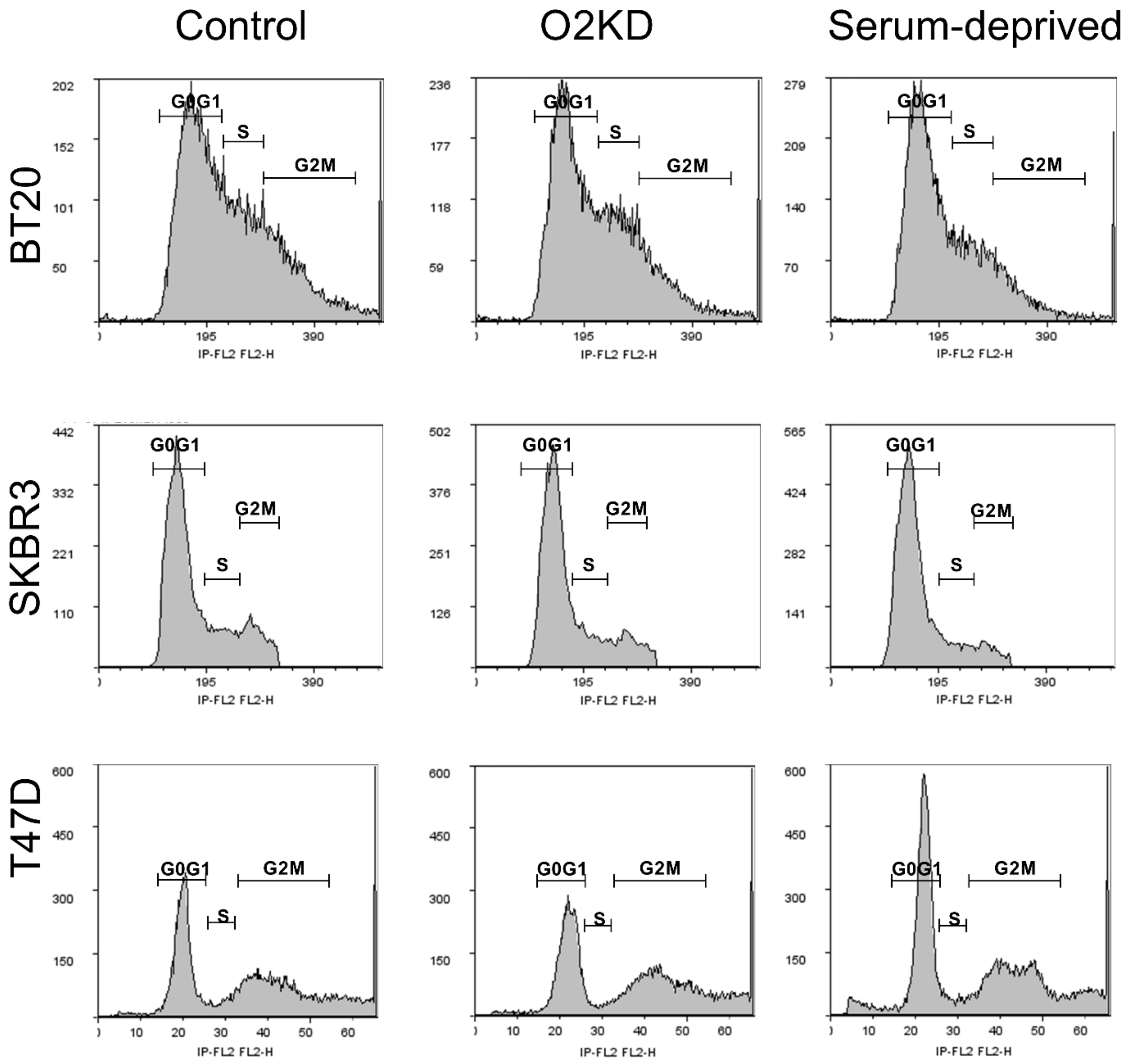

Figure S6. Histogram of DNA content (propidium iodide, PI) versus number of cell counts (cell number), obtained by flow cytometry. BT20, SKBR3 and T47D cells were transfected with esiOrai2 (O2KD) or non-specific siRNA (Control), as indicated, or were serum deprived. Forty-eight hours later, cell cycle analysis through PI staining and following flow cytometry was performed as described in “Materials and Methods”.

Figure 1a: Orai1

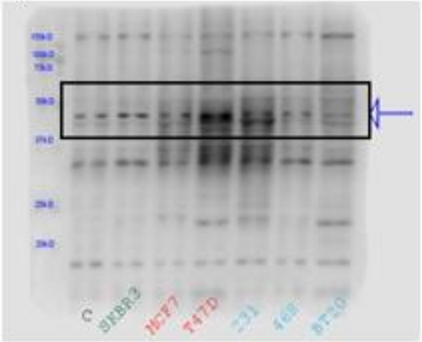

Figure 1a: actin

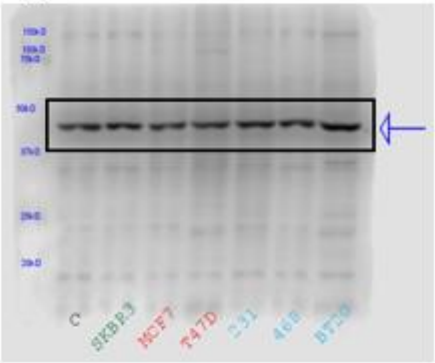

Figure 1b: Orai2

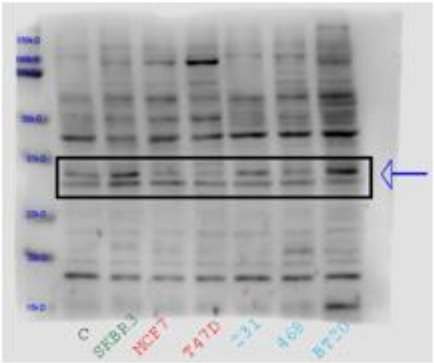

Figure 1b: actin

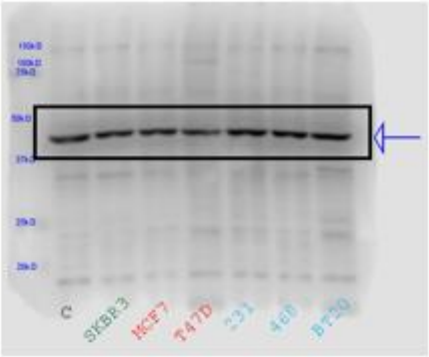

Figure 2a,2f and 2k: Orai2

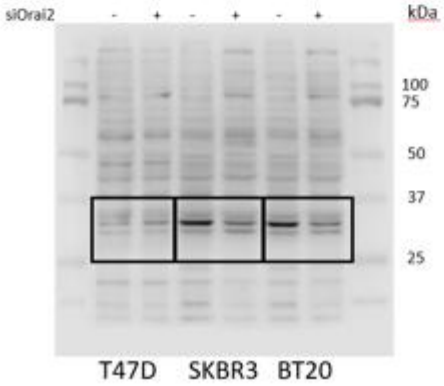

Figure 2a,2f and 2k: actin

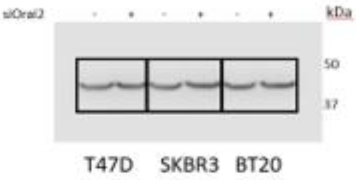

Figure 2S: PMCA

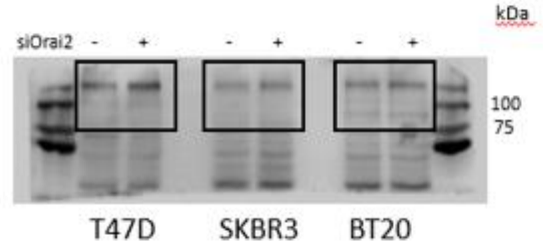

Figure 2S: Orai2

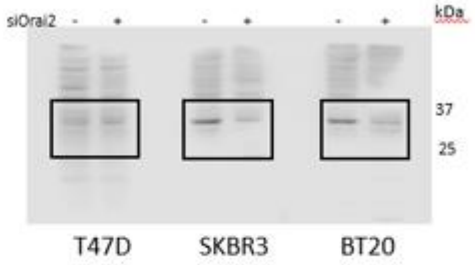

Figure 3S: STIM1

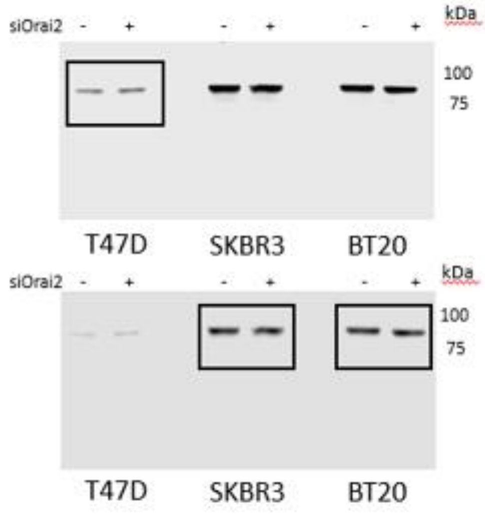

Figure 3S: STIM2

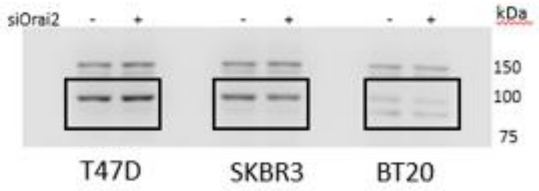

Figure 3S: Orai3

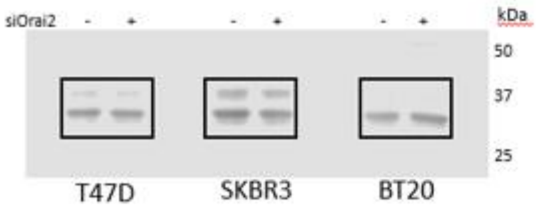

Figure 3S: actin

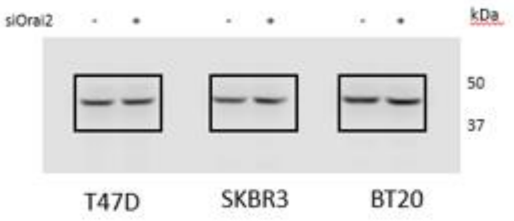

Figure 3S: orai1

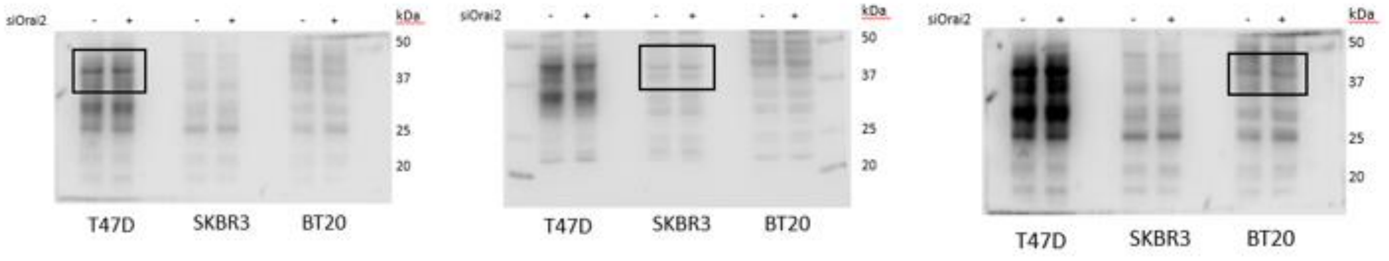

Figure S7. Original Images for Blots/Gels. Uncropped Western blot figures of Figure 1a, 1b, 2a, 2f, 2k, 2S and 3S.

Table S1. Total number of cells analyzed in Figure 3**.**

$[Ca^{2+}]_o$	Control				O2KD			
	0 mM		1 mM		0 mM		1 mM	
CCh (μM)	10	300	10	300	10	300	10	300
BT20	148	101	158	133	162	103	157	129
SKBR3	93	82	165	119	97	81	158	120
T47D	135	140	140	131	157	147	144	126
